# Neurodevelopmental outcomes following late and moderate prematurity: a population-based cohort study

**DOI:** 10.1136/archdischild-2014-307684

**Published:** 2015-04-01

**Authors:** Samantha Johnson, T Alun Evans, Elizabeth S Draper, David J Field, Bradley N Manktelow, Neil Marlow, Ruth Matthews, Stavros Petrou, Sarah E Seaton, Lucy K Smith, Elaine M Boyle

**Affiliations:** 1 Department of Health Sciences, University of Leicester, Leicester, UK; 2 Department of Academic Neonatology, Institute for Women's Health, University College London, London, UK; 3 Division of Health Sciences, Warwick Medical School, University of Warwick, Coventry, UK

**Keywords:** Neurodevelopment, Neonatology

## Abstract

**Objective:**

There is a paucity of data relating to neurodevelopmental outcomes in infants born late and moderately preterm (LMPT; 32^+0^–36^+6^ weeks). This paper present the results of a prospective, population-based study of 2-year outcomes following LMPT birth.

**Design:**

1130 LMPT and 1255 term-born children were recruited at birth. At 2 years corrected age, parents completed a questionnaire to assess neurosensory (vision, hearing, motor) impairments and the Parent Report of Children's Abilities-Revised to identify cognitive impairment. Relative risks for adverse outcomes were adjusted for sex, socio-economic status and small for gestational age, and weighted to account for over-sampling of term-born multiples. Risk factors for cognitive impairment were explored using multivariable analyses.

**Results:**

Parents of 638 (57%) LMPT infants and 765 (62%) controls completed questionnaires. Among LMPT infants, 1.6% had neurosensory impairment compared with 0.3% of controls (RR 4.89, 95% CI 1.07 to 22.25). Cognitive impairments were the most common adverse outcome: LMPT 6.3%; controls 2.4% (RR 2.09, 95% CI 1.19 to 3.64). LMPT infants were at twice the risk for neurodevelopmental disability (RR 2.19, 95% CI 1.27 to 3.75). Independent risk factors for cognitive impairment in LMPT infants were male sex, socio-economic disadvantage, non-white ethnicity, preeclampsia and not receiving breast milk at discharge.

**Conclusions:**

Compared with term-born peers, LMPT infants are at double the risk for neurodevelopmental disability at 2 years of age, with the majority of impairments observed in the cognitive domain. Male sex, socio-economic disadvantage and preeclampsia are independent predictors of low cognitive scores following LMPT birth.

**What is already known on this topic**

- School-aged children born late and moderately preterm are at significantly increased risk for adverse neurodevelopmental outcomes compared with term-born peers.
- Large prospective population-based studies of outcomes in infancy are needed.

**What this study adds**

- Two-year-old children born late and moderately preterm are at double the risk for neurodevelopmental disability compared with term-born peers.
- Risk factors for cognitive impairment include male sex, socio-economic disadvantage, non-white ethnic origin, preeclampsia and not receiving breast milk at discharge.

## Introduction

Preterm birth rates (<37^+0^ weeks) have increased significantly in recent decades, largely due to an increase in late (34^+0^–36^+6^ weeks) and moderately preterm (32^+0^–33^+6^ weeks) deliveries.^1^ Long-term outcomes for late and moderately preterm (LMPT) infants remain poorly characterised although they account for up to 84% of all preterm births.^2^ Compared with term-born peers, increasing numbers of reports indicate that children born at late and/or moderately preterm gestations are at increased risk for health and developmental sequelae,^3–5^ cognitive deficits,^6–8^ learning difficulties^9–13^ and behaviour problems^8^ ^14^ at school age; however, some studies have reported no differences compared with term-born controls.^15^ ^16^

To allow reliable yet early detection of neurodevelopmental sequelae, assessment at 2 years of age is recommended.^17^ Reports of neurodevelopmental outcomes during the first 2 years of life are relatively scarce and have produced conflicting results.^18^ Some have reported an excess of neuromotor, sensory and cognitive impairments in late preterm infants,^19–24^ while others have found no significant differences after adjustment for confounders or correction for prematurity.^21^ ^23^ ^25^ Given the paucity of research to date, several authors have asserted that large prospective population-based studies are needed to estimate the long-term impact of LMPT birth.^26^ ^27^

In this paper we report the results of a prospective population-based study of babies born LMPT compared with term-born controls. The aims of the study were to define neurodevelopmental outcomes at 2 years corrected age and to explore risk factors for adverse cognitive outcomes in LMPT infants.

## Patients and methods

### Population

From September 2009 through December 2010 the mothers of all babies born LMPT (32^+0^–36^+6^ weeks) within a geographically defined region of the East Midlands (UK) were invited to participate in the Late And Moderately preterm Birth Study (LAMBS). This examined births at four maternity centres, a midwifery-led birthing unit and home births during this period. A random sample of babies born at term (37^+0^–42^+6^ weeks) was also recruited during the same time period and in the same geographical region. Eligible term births were selected based on random sampling of dates and times of birth of babies in the same area during the previous year. In addition, mothers of all term-born multiples were invited to participate. Infants with major congenital anomalies were excluded from the present analyses.

### Procedure

The study was approved by Derbyshire NHS Research Ethics Committee (Ref 09/H0401/25). Research midwives obtained informed consent from mothers during their postnatal stay; home visits were arranged for mothers discharged shortly after delivery. Mothers participated in a semi-structured interview after birth and obstetric and neonatal data were collected from mothers’ and infants’ medical records at discharge. Follow-up questionnaires were completed at 2 years corrected age.

### Measures

Mothers were asked about demographic characteristics including ethnicity and language. To quantify socio-economic status (SES), a composite SES-Index score was computed using five proxy variables that measured mothers’ occupation, education, social support, income and wealth. Total SES-Index scores (range 0–12) were used to define three socio-economic risk categories: low (scores 0–2), moderate (scores 3–5) and high (scores ≥6) (see the online supplementary appendix).

Obstetric data collected included maternal chronic health conditions, smoking and recreational drug use during pregnancy, preeclampsia, maternal infection during pregnancy, pre-labour rupture of membranes, antenatal corticosteroids, induction of labour, mode of delivery, raised C-reactive protein (CRP) during delivery and antenatal umbilical Doppler studies. Neonatal data items included sex, gestation, birth weight, small for gestational age (SGA; fetal weight <3rd percentile for sex and gestation using customised antenatal growth charts^28^), respiratory support, hypoglycaemia (blood glucose <2 mmol/L), jaundice requiring phototherapy, antibiotic administration, cranial ultrasound and MRI findings, and feeding at discharge.

At 2 years corrected age, cognitive development was assessed using the Parent Report of Children's Abilities-Revised (PARCA-R).^29^ Scores for non-verbal cognition (NVC; range 0–34) and expressive language (range 0–124) were computed and a total parent report composite (PRC; range 0–158) score derived. PARCA-R scores are strongly correlated with scores on gold standard developmental tests.^29–31^ To identify moderate/severe cognitive impairment, a cut-off score corresponding with PRC scores <2.5th percentile in the term reference group was identified (PRC score <35). Where children had ≤4 missing NVC items (LMPT, n=40; term, n=44), these were substituted with the child's average NVC item score and the PRC score was computed. For 21 non-English speaking children in whom the language scale was not completed, a NVC score <22 corresponding with NVC scores <2.5th percentile of the term reference group was used to classify impairment. Cognitive impairment was not classified for six children with substantial missing PARCA-R data.

Parents were asked whether their child had non-febrile seizures over the past year and whether s/he was currently taking anticonvulsant medication. Parents were also asked whether their child had a diagnosis of cerebral palsy (CP) and were asked to rate their child's vision, hearing and gross motor function (irrespective of CP); forced-choice answers corresponding with criteria for classifying health status following preterm birth^17^ were used to identify the severity of impairment (none, mild, moderate, severe) within each domain. Children with a moderate/severe vision (blind/vision uncorrected with aids), hearing (deaf/hearing uncorrected with aids) or gross motor impairment (non-ambulant/requires assistance to walk) were classified with neuromotor/sensory impairment. These were combined with cognitive impairment to provide a composite measure of neurodevelopmental disability defined as moderate/severe impairment in one or more of vision, hearing, gross motor or cognitive function.

### Statistical analyses

Baseline socio-demographic characteristics were compared between the term and LMPT groups using percentages (χ^2^ test) and means (t test) as appropriate. Neurodevelopmental outcomes were compared between term and LMPT infants both crude and with adjustment for major confounders (sex, SES and SGA) using sandwich estimators to account for clustering of outcomes within multiple births. Sampling weights were used to account for the over-sampling of multiple births among the term group. For binary outcomes, differences between groups were quantified using relative risks obtained using Poisson regression. For continuous outcomes, the mean difference (95% CI) between groups was estimated using linear regression models. PARCA-R scores were converted to z scores using the mean (SD) of the term-born reference group to compare effect sizes across scales. Given the high prevalence of cognitive problems, univariable predictors of cognitive impairment were analysed using Poisson regression. A multivariable model was then constructed to identify independent risk factors using sandwich estimators to account for clustering of outcomes within multiple births. Backwards selection was used with all variables in the univariable analyses entered into the model and dropping out the least significant variable until all had p<0.05; all of the dropped variables were then entered in turn into this preliminary model and included if p<0.05.

## Results

### Population

**Figure 1:**
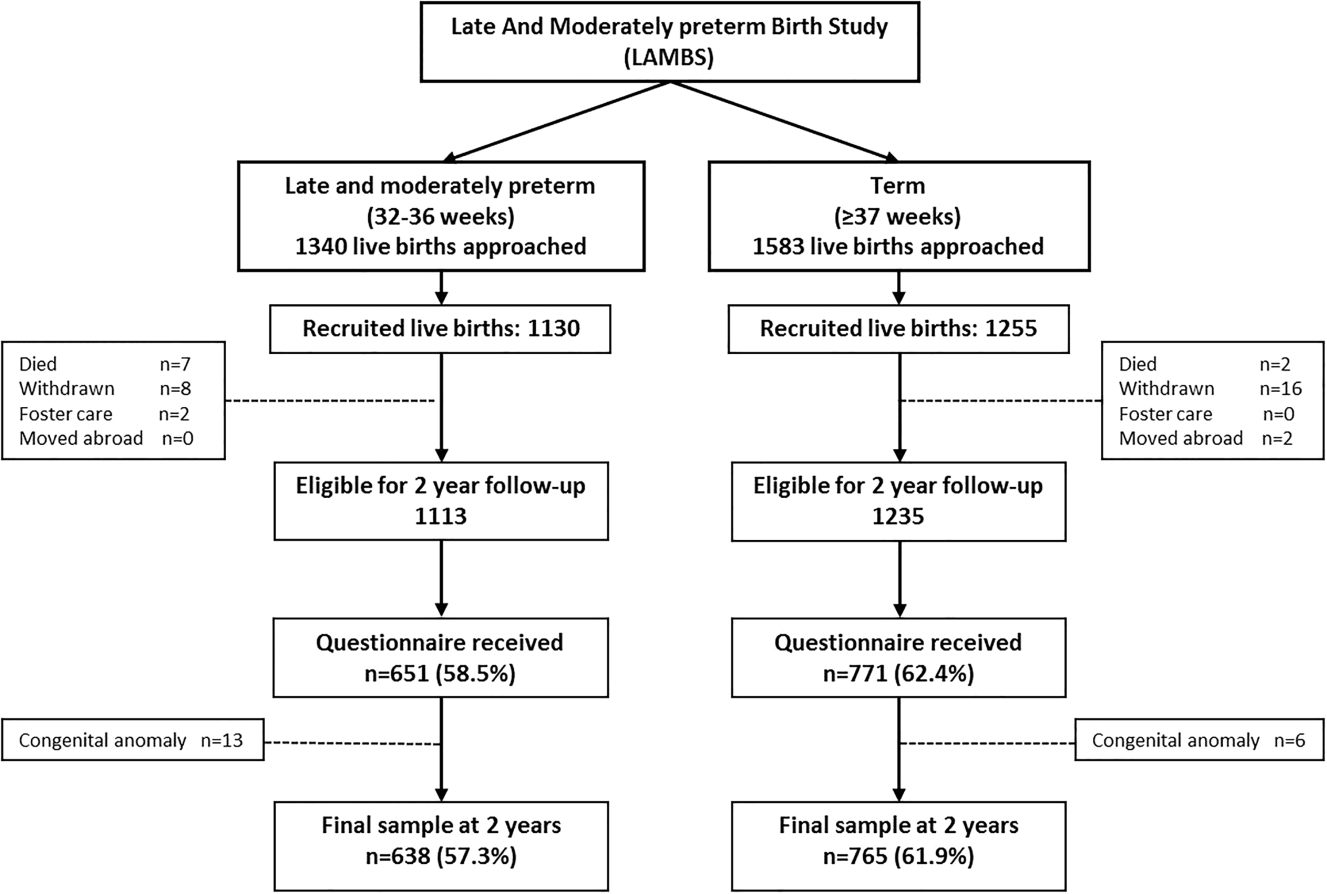
Recruitment, follow-up rates and ascertainment of 2-year outcome data for late and moderately preterm infants and term-born controls.

In total, 1130 LMPT and 1255 controls were recruited. Questionnaires were received for 59% of LMPT and 62% of term-born infants. After exclusion of infants with major congenital anomalies, the final sample comprised 638 (57%) LMPT infants and 765 (62%) controls (figure 1). The characteristics of both groups are shown in table 1. Mothers of LMPT infants were significantly more likely to have high socio-economic risk and LMPT infants were more likely to be born SGA (table 1).

**Table 1:** Baseline socio-demographic characteristics of mothers and their LMPT and term-born infants assessed at 2 years corrected age

Variable	Term	LMPT	p Value
Infants, n	765	638	
Gestational age			
Mean (SD), weeks	39.3 (1.4)	34.9 (1.2)	–
32–33 weeks, n (%)	–	87 (13.6%)	–
34–36 weeks, n (%)	–	551 (86.4%)	–
37–38 weeks, n (%)	241 (31.5%)	–	–
39–40 weeks, n (%)	357 (46.7%)	–	–
41–42 weeks, n (%)	167 (21.8%)	–	–
Multiple birth			
n (%)	151 (19.7)	107 (16.8)	–
Birth weight, g			
Mean (SD)	3322 (535)	2435 (502)	–
Small for gestational age (SGA)*			
n (%)	48 (6.3)	67 (10.5)	0.004
Male sex			
n (%)	384 (50.2)	343 (53.8)	0.18
Corrected age at assessment			
Mean (SD)	24.6 (1.1)	24.6 (1.0)	0.41
**Mothers**	**N=690**	**N=587**	**p Value**
Age			
<20 years, n (%)	16 (2.3)	19 (3.2)	0.56
20–24 years, n (%)	96 (13.9)	86 (14.7)	0.68
25–29 years, n (%)	181 (26.2)	175 (29.9)	–
30–34 years, n (%)	209 (30.3)	192 (32.8)	0.73
≥35 years, n (%)	188 (27.3)	114 (19.5)	0.003
Ethnic group			
White, n (%)	569 (82.5)	461 (78.5)	–
Mixed, n (%)	7 (1.0)	12 (2.0)	0.118
Asian or Asian British, n (%)	77 (11.2)	86 (14.7)	0.057
Black or Black British, n (%)	30 (4.4)	21 (3.6)	0.62
Chinese or other, n (%)	7 (1.0)	6 (1.0)	0.92
Unknown, n (%)	0 (0.0)	1 (0.2)	–
English not first language			
n (%)	85 (12.3)	76 (13.0)	0.66
SES-Index			
Low risk, n (%)	339 (49.1)	256 (43.6)	–
Medium risk, n (%)	209 (30.3)	184 (31.4)	0.24
High risk, n (%)	142 (20.6)	147 (25.0)	0.028

*SGA classified as fetal weight <3rd percentile for sex and gestation using customised antenatal growth charts.^28^

SES-Index refers to socio-economic risk category derived from a composite measure of five indices of socio-economic risk (see the online supplementary appendix).

LMPT, late and moderately preterm.

The characteristics of non-responders have been described previously.^32^ Non-responding mothers were younger, more likely to be non-white, non-English speaking and single parents, to have a lower occupational status and educational qualifications, to be struggling financially and to have poorer health than responders.

### Neuromotor and sensory outcomes

LMPT children were at significantly increased risk for neuromotor/sensory impairment (1.6% vs 0.3%; RR 4.89, 95% CI 1.07 to 22.25; table 2). The prevalences of hearing, vision and gross motor impairments were each 0.3–0.5% higher in LMPT infants than in controls and CP was more common in term-born infants (0.5% vs 0%), but the low prevalence of these disorders precluded assessment of the significance of group differences in individual domains. There was no significant excess of seizures or use of anticonvulsant medication in LMPT infants.

**Table 2:** Neurodevelopmental outcomes at 2 years corrected age among late and moderately preterm (LMPT) infants and term-born controls

Neurodevelopmental outcome	Moderately preterm	Late preterm	All LMPT	Term	Difference LMPT vs term*			
	(n=87)	(n=551)	(n=638)	(n=765)	Unadjusted RR (95% CI)	p Value	Adjusted† RR (95% CI)	p Value
Neurological outcomes								
Seizures, n (%)	0	2 (0.4)	2 (0.3)	1 (0.1)	1.96 (0.17 to 21.61)	0.58	–	–
Prescribed anticonvulsants, n (%)	0	1 (0.2)	1 (0.2)	2 (0.3)	0.49 (0.04 to 5.39)	0.56	–	–
Neuromotor and sensory impairment								
Cerebral palsy, n (%)	0	0	0	4 (0.5)	–	–	–	–
Hearing impairment, n (%)	0	3 (0.5)	3 (0.5)	0 (0.0)	–	–	–	–
Vision impairment, n (%)	0	2 (0.4)	2 (0.3)	0 (0.0)	–	–	–	–
Gross motor impairment, n (%)	0	5 (0.9)	5 (0.8)	2 (0.3)	2.44 (0.47 to 12.57)	0.29	–	–
Neuromotor/sensory impairment‡, n (%)	0	10 (1.8)	10 (1.6)	2 (0.3)	4.89 (1.07 to 22.25)	0.04	–	–
Cognitive developmen**t**§					Mean difference (95% CI)		Mean difference (95% CI)	
Non-verbal cognition, mean (SD)	27.1 (4.3)	27.6 (4.5)	27.5 (4.4)	28.0 (3.4)	−0.59 (−1.03 to −0.13)	0.01	−0.49 (−0.94 to −0.03)	0.04
Expressive language, mean (SD)	58.9 (32.3)	61.7 (34.0)	61.3 (33.7)	66.4 (31.7)	−5.14 (−8.89 to −1.39)	0.007	−3.96 (−7.62 to −0.31)	0.03
Total PRC score, mean (SD)	86.0 (34.5)	89.3 (36.2)	88.9 (36.0)	94.5 (33.3)	−5.80 (−9.78 to −1.82)	0.004	−4.49 (−8.36 to −0.62)	0.02
					RR (95% CI)		RR (95% CI)	
Cognitive impairment§, n (%)	4 (4.7)	36 (6.6)	40 (6.3)	18 (2.4)	2.66 (1.53 to 4.62)	0.001	2.09 (1.19 to 3.64)	0.01
Neurodevelopmental disability¶, n (%)	4 (4.7)	40 (7.3)	44 (6.9)	19 (2.5)	2.37 (1.38 to 4.08)	0.002	2.19 (1.27 to 3.75)	0.004

*Analyses were weighted to account for over-sampling of term-born multiples.

†Group differences adjusted for sex, SES-Index and SGA.

‡Neuromotor/sensory impairment is classified where a child has a moderate/severe impairment in any one of hearing, vision or motor function.

§Cognitive development was measured using the Parent Report of Children's Abilities-Revised and is defined as a PRC score of <35.

¶Neurodevelopmental disability is defined as a moderate/severe impairment in any one of hearing, vision, motor or cognitive function.

PRC, parent report composite; SGA, small for gestational age.

### Cognitive outcomes

**Figure 2:**
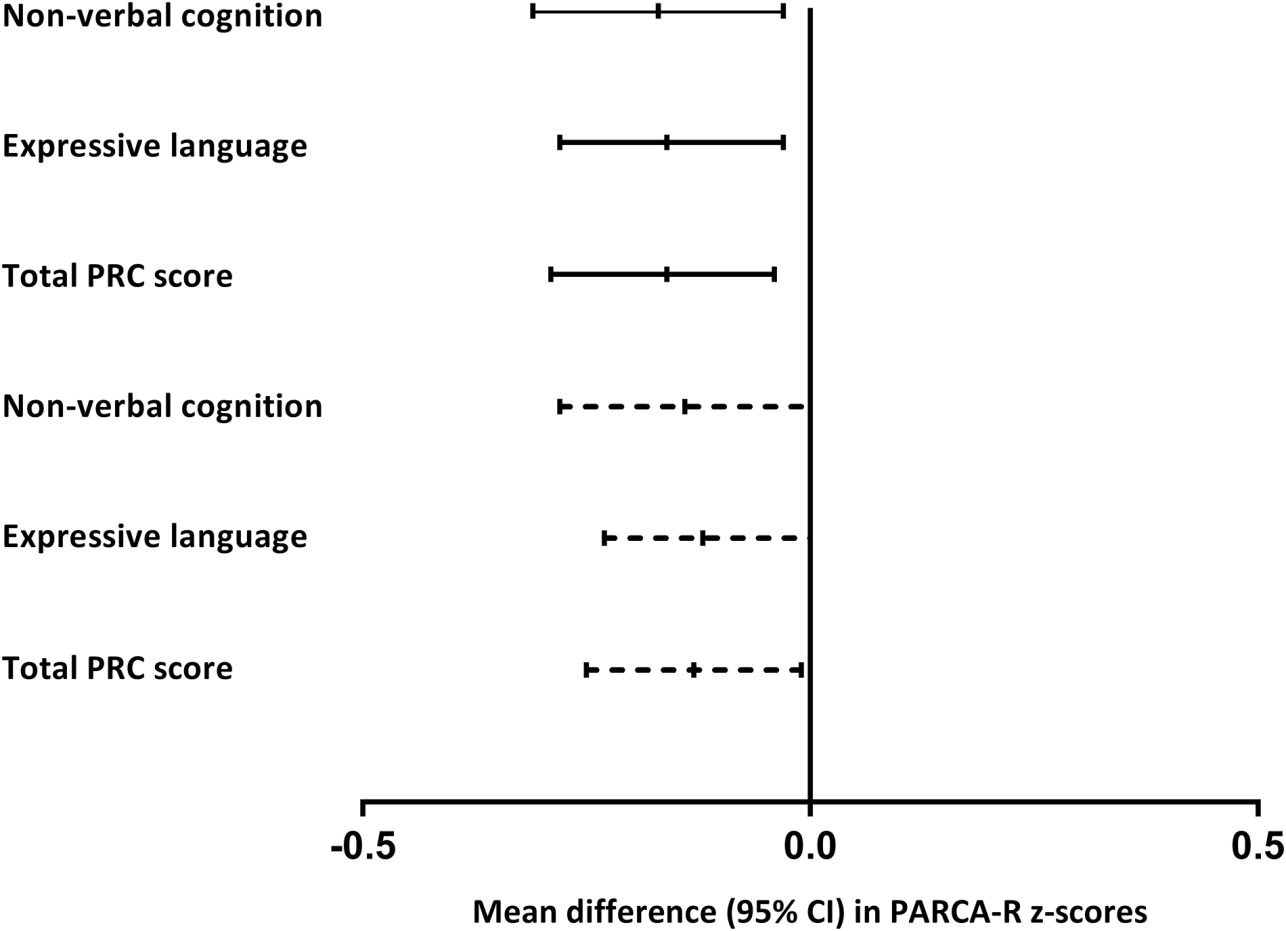
Mean difference (95% CI) in Parent Report of Children's Abilities-Revised (PARCA-R) z scores between late and moderately preterm (32–36 weeks gestation) and term-born (37–42 weeks gestation) infants. z Scores were calculated using the mean (SD) of the term reference group. Solid lines represent crude differences and dashed lines represent differences adjusted for sex, socio-economic status and small for gestational age (SGA) status. PRC, parent report composite.

LMPT children had significantly lower mean scores than controls on all PARCA-R scales (table 2), which equated to a 0.14–0.15 SD deficit in both language and non-verbal cognition (figure 2). LMPT infants were significantly more likely to have moderate/severe cognitive impairment than controls (6.3% vs 2.4%; adjusted RR 2.09, 95% CI 1.19 to 3.64). Among LMPT infants, boys were significantly more likely to have moderate/severe impairment than girls (10.5% vs 1.4%; RR 7.77, 95% CI 2.78 to 21.50), but there was no significant sex difference among controls (3.2% vs 1.6%; RR 2.01, 95% CI 0.75 to 5.30).

### Neurodevelopmental disability

LMPT infants were at significantly increased risk for moderate/severe neurodevelopmental disability (6.9% vs 2.5%; adjusted RR 2.19, 95% CI 1.27 to 3.75; table 2). Of 44 LMPT infants with disability, 40 (91%) had cognitive impairment compared with 18 of 19 (95%) controls with disability.

### Risk factors for cognitive impairment in LMPT infants

Univariable analyses revealed that LMPT infants born to mothers aged ≥35 years, of a non-white ethnic origin, with medium or high socio-economic risk, pre-pregnancy hypertension or preeclampsia were more likely to have moderate/severe cognitive impairment (table 3). Of the neonatal factors examined, only male sex, hypothermia (<36°C) and not receiving breast milk at discharge were significantly associated with moderate/severe cognitive impairment. Multivariable regression models identified five independent risk factors for cognitive impairment in LMPT infants (table 3): male sex exerted the greatest effect (RR 7.04, 95% CI 2.52 to 19.67), while high socio-economic risk, non-white ethnic origin, preeclampsia and not receiving breast milk at discharge were also independent predictors.

**Table 3:** Associations between demographic, obstetric and neonatal factors and cognitive impairment at 2 years corrected age in LMPT infants

Variable	Cognitive impairment (n=40)		Univariable analyses	p Value	Multivariable analyses	p Value
	Obstetric/neonatal risk factor present, n (%)‡	Obstetric/neonatal risk factor absent, n (%)‡	RR (95% CI)		RR (95% CI)	
Obstetric risk factors						
Mother’s age						
<20 years	1 (5.0)	39 (6.3)	1.31 (0.16 to 10.17)	0.793	–	–
20–24 years	8 (9.0)	32 (5.8)	2.36 (0.88 to 6.32)	0.086	–	–
25–29 years	7 (3.8)	33 (7.3)	Baseline	–	–	–
30–34 years	11 (5.1)	29 (6.9)	1.33 (0.52 to 3.37)	0.544	–	–
35+ years	13 (10.4)	27 (5.3)	2.73 (1.12 to 6.67)	0.027	–	–
Non-white ethnic group	13 (10.1)	27 (5.4)	1.88 (1.00 to 3.55)	0.050	2.06 (1.10 to 3.83)	0.023
Non-English speaking at home	6 (7.5)	33 (6.1)	1.23 (0.53 to 2.84)	0.632	–	–
SES-Index						
Low risk	8 (2.8)	32 (9.1)	Baseline	–	–	–
Medium risk	18 (9.2)	22 (4.8)	3.26 (1.44 to 7.35)	0.004	2.86 (1.24 to 6.57)	0.013
High risk	14 (9.0)	26 (5.3)	3.19 (1.36 to 7.43)	0.007	2.36 (1.02 to 5.48)	0.046
Conceived via infertility treatment	0	40 (6.9)	–	–	–	–
Pre-pregnancy diagnosed diabetes	1 (4.6)	39 (6.4)	0.72 (0.10 to 4.99)	0.735	–	–
Pre-pregnancy diagnosed hypertension	3 (20.0)	37 (6.0)	3.36 (1.16 to 9.69)	0.025	–	–
Smoked during pregnancy*	11 (8.6)	29 (5.7)	1.50 (0.76 to 2.94)	0.238	–	–
Drank alcohol during pregnancy†	18 (6.3)	22 (6.3)	1.00 (0.54 to 1.86)	0.997	–	–
Recreational drugs used during pregnancy‡	1 (8.3)	39 (6.3)	1.33 (0.22 to 7.86)	0.750	–	–
Preeclampsia	12 (12.8)	28 (5.2)	2.47 (1.25 to 4.87)	0.009	2.51 (1.33 to 4.70)	0.004
Infection (+culture) during pregnancy	1 (11.1)	39 (6.2)	1.79 (0.27 to 11.66)	0.544	–	–
Gestational diabetes	3 (12.5)	36 (5.9)	2.11 (0.71 to 6.26)	0.176	–	–
Pre-labour rupture of membranes >24 h	7 (5.7)	33 (6.5)	0.88 (0.39 to 1.95)	0.745	–	–
Antenatal corticosteroids given	8 (4.6)	31 (6.8)	0.68 (0.31 to 1.46)	0.320	–	–
Labour induced	9 (6.6)	30 (6.0)	1.10 (0.53 to 2.28)	0.800	–	–
Raised CRP during labour (>5 mg/L)	1 (4.2)	38 (6.5)	0.64 (0.09 to 4.19)	0.645	–	–
Normal vaginal delivery	20 (6.2)	20 (6.4)	0.98 (0.53 to 1.82)	0.952	–	–
Absent or reversed end diastolic flow	2 (7.7)	38 (6.2)	1.23 (0.30 to 4.96)	0.766	–	–
Neonatal risk factors						
Male	36 (10.5)	4 (1.4)	7.74 (2.77 to 21.55)	<0.001	7.04 (2.52 to 19.67)	<0.001
Gestational age						
36 weeks	22 (8.0)	18 (5.0)	Baseline	–	–	–
35 weeks	6 (3.6)	34 (7.2)	0.45 (0.18 to 1.10)	0.080	–	–
34 weeks	8 (7.3)	32 (6.1)	0.91 (0.40 to 2.01)	0.807	–	–
33 weeks	3 (6.3)	37 (6.3)	0.78 (0.24 to 2.48)	0.671	–	–
32 weeks	1 (2.6)	39 (6.5)	0.33 (0.04 to 2.39)	0.271	–	–
Multiple birth	4 (3.7)	36 (6.8)	0.55 (0.16 to 1.85)	0.333	–	–
Small for gestational age§	–	–	–	–	–	–
>10th centile	35 (6.2)	5 (6.8)	Baseline	–	–	–
>3rd and ≤10th centile	2 (5.0)	38 (6.4)	0.80 (0.19 to 3.24)	0.759	–	–
≤3rd centile	3 (9.1)	37 (6.1)	1.46 (0.47 to 4.55)	0.511	–	–
Resuscitated at birth	8 (7.1)	32 (6.1)	1.16 (0.54 to 2.43)	0.702	–	–
Any respiratory support received¶	6 (7.1)	34 (6.2)	1.14 (0.49 to 2.67)	0.755	–	–
Intracranial abnormality**	0 (0)	40 (6.3)	–	–	–	–
Jaundice requiring phototherapy	2 (4.1)	36 (6.6)	0.62 (0.15 to 2.52)	0.502	–	–
Hypoglycaemia (<2 mmol/L)	4 (9.3)	36 (6.1)	1.53 (0.57 to 4.11)	0.396	–	–
Hypothermia (<36°C)	7 (13.0)	33 (5.7)	2.29 (1.06 to 4.93)	0.035	–	–
Antibiotics given	16 (7.3)	24 (5.8)	1.27 (0.68 to 2.34)	0.445	–	–
Any breast milk at discharge††	17 (4.3)	23 (9.5)	0.46 (0.24 to 0.84)	0.011	0.52 (0.28 to 0.95)	0.032

Data are shown for all independent variables entered in univariable analyses, and for factors that were significant independent predictors in multivariable analyses.

*Smoked during pregnancy is classified as mothers who smoked at least one cigarette per day at any time during pregnancy versus <1 cigarette per day; data were missing for two mothers.

†Drank alcohol during pregnancy is classified as mothers who drank any alcohol at any time during pregnancy versus no alcohol.

‡Recreational drugs used during pregnancy was classified for one or more instances of drug use at any time during pregnancy.

§Fetal weight for sex and gestation classified using customised fetal growth charts.^28^

¶Any respiratory support includes infants who were ventilated or received non-invasive respiratory support.

**Intra-cranial abnormality includes grade III or IV intra-ventricular haemorrhage, periventricular leukomalacia and grade II or III neonatal encephalopathy.

††Includes breast milk fed by any method. Data were missing for three mothers for gestational diabetes.

‡‡n (%) of infants with cognitive impairment where the obstetric/neonatal risk factor is present (column 2) and absent (column 3).

CRP, C-reactive protein; LMPT, late and moderately preterm.

## Discussion

The adverse effects of LMPT birth are already evident at 2 years of age, with LMPT infants having double the risk of neurodevelopmental disability compared with term-born controls. The significant increase in neurodevelopmental disability was almost entirely due to cognitive deficits. Among LMPT infants, mean cognitive and language scores were 0.15 SD lower than among controls, which is equivalent to a 2.3-point deficit in standardised IQ scores. Similar to very preterm infants, this may be indicative of aberrant brain development.^33^ Substantial neurodevelopment occurs in the third trimester, including a fourfold increase in cortical volume, increased myelination and rapid cerebellar development.^34–36^ Even at LMPT gestations, preterm birth may impede the normal trajectory of brain development.^37^

Cognitive deficits of a similar magnitude have been reported in school-aged children born late preterm, although in some studies these differences were not significantly different from controls.^6–8^ ^15^ ^16^ Comparisons between studies are problematic given the heterogeneity in population characteristics, age at assessment and outcome measures.^38^ However, Nepomnyaschy *et al*^21^ reported that late preterm infants had significantly lower cognitive and language scores at 2 years, but there was a significant group difference only in language after adjustment for confounders. Woythaler and colleagues^20^ also reported significantly lower cognitive scores at 2 years in the same cohort. In contrast, smaller studies have not found significant group differences at this age, particularly where corrected age has been applied.^23^ ^39–41^ Since corrected age was used to time assessments in the present study, our findings in terms of both significantly lower mean scores and higher prevalence of impairment are notable. Although the prevalence of neuromotor and sensory impairment was low, rates were 0.3–0.5% higher in the LMPT group. We were unable to assess the significance of group differences in individual domains and the 95% CI for composite neurosensory impairment was wide. However, our results are borne out by the findings of record-linkage studies that have reported a significant excess of neurological sequelae and CP.^19^ ^37^ ^42^

Few studies have investigated antecedents of adverse outcomes in LMPT infants. In the present study, the strongest risk factor for low cognitive scores was male sex: LMPT boys were at sevenfold increased risk compared with LMPT girls. Among males, LMPT birth conferred a greater risk of moderate/severe impairment compared to controls (10.5% vs 3.2%), while rates among female LMPT infants and controls were similar (1.4% vs 1.6%). The male disadvantage in neurodevelopmental outcomes is well documented in preterm cohorts and the interaction between sex and gestation may explain much of the disadvantage observed here among our LMPT population. As expected, socio-demographic factors were also markers of adverse outcomes; the additive impact of socio-economic factors on long-term outcomes has previously been reported in this population.^11^ ^43^

Preeclampsia was also identified as an independent risk factor and has been associated with long-term cognitive and behavioural sequelae in general population samples,^44–46^ and it has been suggested that adverse behavioural outcomes in late preterm infants may be associated with maternal hypertensive disease.^47^ Worsening symptoms of preeclampsia frequently lead to delivery by induction or caesarean section. In such cases the maternal and fetal risks must be weighed against the long-term effects of prematurity. Further research is needed to disentangle the relative contribution of hypertensive disease and prematurity to long-term outcomes.

It was noted that lack of continuing provision of breast milk at discharge was associated with moderate/severe cognitive impairment. Among extremely preterm infants this has been identified as an independent risk factor for autism and psychiatric disorders.^48^ ^49^ The mechanisms underlying this association are unclear; the relationship may reflect socio-economic disadvantage, parental aspirations, early attachment, neurological difficulties or a direct role of breast milk in neuronal development.^49^

### Strengths and limitations

The present study addresses the growing need for large, population-based investigations of outcomes following LMPT birth. Data were collected from a birth cohort spanning a wide geographical region of the East Midlands of England and the prospective nature enabled an investigation of risk factors for adverse outcomes including neonatal, antenatal and maternal lifestyle factors. Neurodevelopmental outcomes were classified using standard criteria for defining health status at 2 years^17^ and contemporaneous reference data were used to define cut-offs for cognitive impairment as recommended in follow-up studies.^50^ ^51^ Group differences in outcomes were also investigated after adjustment for important confounders.

The major limitation of this study was the response rate at 2 years and the selective dropout of mothers with greater socio-demographic risk. This may have resulted in an underestimation of the true prevalence of adverse outcomes; however, the factors affecting non-response were the same in both groups and thus the relative risks reported are likely to be reflective of the total population. The size of this study necessitated the use of parent questionnaires as outcome measures. Although these may be considered less preferable than developmental tests, well-validated tools were used where possible. In particular, the use of parent reports may have resulted in underestimation of the true prevalence of CP as this may be diagnosed later in childhood, particularly for infants with mild neuromotor signs. Longer term follow-up is needed to determine their prognostic value for later functional outcomes. Despite the sizeable cohort recruited, the study was powered to detect a difference in cognitive impairment between two groups (LMPT vs term). As such, we were unable to assess the statistical significance of group differences in neuromotor and sensory impairments and there was insufficient statistical power to explore a dose–response relationship with gestation age at birth.

## Conclusions

Prematurity remains one of the major causes of infant mortality and lifelong morbidity worldwide. We have demonstrated that babies born at 32–36 weeks of gestation are at double the risk for neurodevelopmental disability at 2 years of age, with the vast majority of identified impairments in the cognitive domain. Given the size of the LMPT population, even the small increases in impaired outcomes observed in the present study may have significant long-term public health implications.

## Supplementary Material

Web appendix
