# Supplementary material for: Neurodevelopmental outcomes following late and moderate prematurity: a population-based cohort study
Source: Arch Dis Child Fetal Neonatal Ed. 2015 Apr 1;100(4):F301–8. doi: 10.1136/archdischild-2014-307684 (PMC4484499; doi:10.1136/archdischild-2014-307684)
Supplement: Web appendix [file fetalneonatal-2014-307684-s1.pdf]

APPENDIX: Composition of the Socio-Economic Status Index (SES-Index).

Table A1 shows the composition of the SES-Index score and the contribution of each risk factor to the composite score. Where there were missing data for highest educational qualification, mothers' age at leaving continuous education was used as a proxy measure. For occupation, the risk status of mothers who rated themselves as not in paid employment as a result of as looking after their family was coded using receipt of means-tested benefits as a proxy measure. A total SES-Index score was computed for each mother which was used to classify overall socio-economic risk as low, moderate and high.

Table A1. Composition of the Socio-Economic Status Index (SES-Index)

**Maternal risk indicator**	**Low risk Score=0**	**Mild Score=1**	**Moderate Score=2**	**High Score=3**
**EDUCATION: Highest qualification**	Degree or equivalent	A Level or equivalent	GCSE Grade A-C or equivalent	GCSE ≤Grade D or equivalent
If missing, use age at end of continuous education	*(≥19y)*	*(18y-21y)*	*(16y-17y)*	*(<16y)*
**OCCUPATION: Occupational status**	Class Managerial, professional, or administrative occupations	Intermediate, supervisory, or small employer occupations	Routine or semi-routine occupations	Unemployed or never worked
If housewife, use receipt of means-tested benefits		*Not claiming benefits*		*Claiming benefits*
**SOCIAL SUPPORT: Co-habiting status**	Living with a partner	-	Not living with a partner	-
**INCOME: Car ownership**	Access to ≥1 car in the household	-	No household access to a car	-
**WEALTH: Home ownership**	Home owner/mortgage	-	Rents/part rents/ lives rent free	-
**Total SES-Index score**	**Scores 0-2**	**-**	**Scores 3-5**	**Scores ≥6**
